# The Dixon-Jones Case

**Published:** 1890-04

**Authors:** 


### THE DIXON-JONES CASE.

Most of our readers are doubtless aware of the indictment and trial for manslaughter of Dr. Mary A. Dixon-Jones and her son, Dr. Charles N. Dixon-Jones, of Brooklyn, and of their acquittal after a most thorough, exhaustive, and elaborate trial. A Mrs. Ida Hunt died after an abdominal section in which diseased uterine appendages were removed, and this formed the basis of the indictment and trial.

The *Medical Record*, in its issue of March 1st, editorially says:

> The original indictment of the Grand Jury of King's County was founded upon a series of baseless charges, persistently reiterated in a Brooklyn journal of large circulation, bearing upon unskilful treatment of the case, wilful and culpable neglect of the ordinary obligations of humanity, and dark, mysterious, and even murderous doings in the management of the hospital with which she is connected. All these allegations, absurd as they were, inflamed the public mind against the doctor, almost ruining a hitherto untarnished reputation. When, however, the case came to trial, the real facts were brought out, and an unqualified and triumphant acquittal was the result.

We should be glad, did space permit, to reproduce the *Record's* stirring editorial entire, for it is a masterly exposition of the case, and condemns in fitting words the outrage that was sought to be perpetrated in the name of law and justice. We refer to the case now more particularly to call attention to the imperfect and unseemly manner in which these prosecutions are frequently—nay, generally—brought against reputable physicians, and to suggest that it is high time that both the public press and the lawyers, even in this free American Republic, were more conservative in the exercise of their functions, when a slight mistake, or worse, a brutal blunder, may forever smirch a fair, untarnished reputation—that immortal part without which all else is bestial. But in this case, thanks to Judge Bartlett—a "most learned judge"—and to four or five good witnesses, who dared to do right, this poor, maligned woman and able physician has been permitted to preserve her good name and fame as a sweet inheritance for her noble son, whose manly attitude during this trying ordeal must everywhere command respect.

To Drs. Polk, Morris, Wylie, Coe, and Heintzman, of New York, and Joseph Price, of Philadelphia, the profession owes an everlasting debt of gratitude for their disinterested and able support of the right, which enabled court and jury to reach a just solution of this legal (?) monstrosity, and thus preserve the dignity of two outraged though learned and noble professions.

